# Effects of positive reappraisal and self-distancing on the meaningfulness of everyday negative events

**DOI:** 10.3389/fpsyg.2023.1093412

**Published:** 2023-02-15

**Authors:** Clement Yong Hao Lau, William Tov

**Affiliations:** School of Social Sciences, Singapore Management University, Singapore, Singapore

**Keywords:** meaning in life, situational meaning, positive reappraisal, self-distancing, daily negative experiences

## Abstract

Current work on meaning-making has primarily focused on major negative life events such as trauma and loss, leaving common daily adversities unexplored. This study aimed to examine how utilizing meaning-making strategies such as positive reappraisal and self-distancing (in isolation or in combination) can facilitate an adaptive processing of these daily negative experiences. Overall meaning and facets of meaning (coherence, purpose, and significance/mattering) were assessed at both global and situational levels. Results suggested that positive reappraisal was generally effective for enhancing situational meaning but not under all conditions. Specifically, when negative experiences were high on emotional intensity, reflecting on the experience from a distanced (third-person) perspective enhanced coherence and existential mattering more than engaging in positive reappraisal. However, when negative experiences were low on intensity, distanced reflection led to less coherence and mattering than positive reappraisal. The findings of this study elucidated the importance of examining the multidimensional construct of meaning at the facet level and highlighted the importance of applying different coping strategies to effectively make meaning out of daily negative experiences.

## 1. Introduction

Meaning—a sense of understanding, significance, and purpose—is central to human experience (Baumeister, 1991; Park and Folkman, 1997; Park, 2010). Under the extreme conditions of life in a concentration camp, Frankl (1984, 2011) observed that meaning in life (MIL) was critical for well-being and survival in the face of adversity. Numerous studies have found that meaning is associated with psychological health. For example, MIL is positively associated with happiness (Debats et al., 1993) and life satisfaction (Steger et al., 2008), and negatively related to depressive and anxiety symptoms (Ishida and Okada, 2006). Those who believe their lives are meaningful tend to exhibit a lower incidence of psychological disorders and lesser suicidal ideation than those who believe their lives are meaningless (Owens et al., 2009; Steger and Kashdan, 2009).

As distressing experiences may disrupt one’s sense of meaning (such as whether life continues to be worthwhile), it is important to identify how efforts to restore meaning can help to sustain positive functioning (e.g., Carnelley and Janoff-Bulman, 1992; Davis and Camille, 2000). In the present study, we explored the potential for positive reappraisal and self-distancing (e.g., recalling an experience from a third-person perspective) to extract meaning from daily negative experiences. Further, we examined whether each approach is more or less effective when practiced in combination versus in isolation.

### 1.1. Global meaning and situational meaning

According to the meaning-making model (Park, 2010), there are two levels at which people can experience a sense of meaning: global meaning and situational meaning. Global meaning—or meaning in life—refers to the belief and the sense that the world and one’s place within it are coherent and comprehensible, and that one is progressing toward value-consistent goals. It is derived through individuals’ global beliefs (i.e., assumptions about how the world functions, self and identity, human nature, and relationships; Park, 2017a), and global goals (i.e., life aspirations, values, and strivings; Park, 2010). These beliefs and goals form the frameworks through which people interpret and evaluate their life as whole and their experiences in general (Silberman, 2005). One’s sense of global meaning develops within a broader cultural context (Austin and Vancouver, 1996). As such, it is not surprising to see that culture can exert effects on global meaning (Tweed and Conway, 2006) and meaning-making processes (Neimeyer et al., 2002). Drawing on terror management theory, an individual’s cultural worldview offers a framework for understanding the world and our place in it. As such, heightening the endorsement of one’s cultural worldview helps to provide a sense of meaning that offers symbolic protection against the existential terror of one’s own mortality.

Situational meaning refers to an individual’s interpretation of the importance or significance of a particular experience (i.e., how one construes an event), and its impact on one’s values and beliefs (Park and Folkman, 1997; Lazarus, 2006). As with global meaning, situational meaning may also be associated well-being (Park and Gutierrez, 2012). For example, appraising events as controllable and benign is related to less distress following negative events (Aldwin et al., 2007; Frazier et al., 2011). Although much research has concentrated on the appraisal of negative events and its impact on stress (e.g., Lazarus and Folkman, 1984), few studies have explicitly examined whether such processes enhance or reduce both situational and global meaning. However, examining both in tandem is necessary for a complete understanding of how meaning is experienced holistically (Park, 2017b).

Moreover, several researchers have criticized the use of generic measures of “meaning” or “purpose” in studies of MIL (Leontiev, 2013; Heintzelman and King, 2014; King and Hicks, 2021). There is an emerging consensus that meaning is a multidimensional construct composed of at least three facets (George and Park, 2016, 2017; Martela and Steger, 2016). Firstly, a sense of *coherence*—which entails making sense of and comprehending one’s experiences (Reker and Wong, 2012). Secondly, a sense of *purpose*—which involves the motivation to pursue *valued* life goals (Kasser and Ryan, 1993; McGregor and Little, 1998; Rijavec et al., 2011). Lastly, a sense of *significance* or *mattering* refers to the feeling that one’s life is worth living and that one’s existence is important and of value in the world (George and Park, 2014; King et al., 2016).

Furthermore, while previous studies of the tripartite model (coherence, purpose, and mattering) have focused on global meaning (e.g., Costin and Vignoles, 2020), few have examined facet-level meaning at the situational level (e.g., Tov et al., 2021). However, such work is necessary for a better understanding of how processes that occur at the situational level may contribute to the formation of meaning at the global level. As such, this study sought to examine how meaning can be extracted from negative experiences—at both the global and situational levels. In addition, we also assessed overall meaning and specific facets of meaning.

### 1.2. Effects of negative experiences on meaning

Broadly, negative experiences—from the mundane (e.g., arguments) to the severe (e.g., death of a loved one)—are often associated with a diminished sense of meaning (Krause, 2005; Vohs et al., 2019). When individuals experience a distressing event, the situational meaning of the event (e.g., its impact on current goals, or one’s understanding of what happened and why) can violate their global beliefs. This violation—which usually stems from the individual’s perception of their loss of control, comprehensibility, or predictability of the world (Park and Folkman, 1997; Davis and Camille, 2000; Heine et al., 2006)—may compromise the integrity of their global meaning system (Janoff-Bulman, 1989; Park et al., 2016) and challenge their understanding of themselves and the world. For example, religious individuals may generally believe that God is good and kind. However, following the loss of a significant other, bereaved individuals may interpret death as willed by God’s intention. This could lead them to question God’s character, possess negative feelings toward God, and choose to abandon their faith (Burke et al., 2014). This change in global meaning (e.g., faith) due to the situational meaning (e.g., appraisal of the death of a significant other) highlights that a negative experience can lead to a discrepancy between meaning at both levels.

It is not always the case that changes in situational meaning provoke changes in global meaning. According to the meaning-making model, when there are discrepancies between situational and global meaning, individuals are often motivated to reduce them through the meaning-making process (Janoff-Bulman and McPherson Frantz, 1997; Park, 2010). This can involve an attempt to cognitively process and understand the negative situation in a different way, or review and rework their beliefs so that the interpretation of the situation aligns with their global meaning (Klinger, 1998; Lepore et al., 2000). Of particular relevance to the present study is the process of assimilation. Through assimilation, individuals may change how they appraise the situation (i.e., situational meaning) so that it is aligned to their global assumptions (Park, 2010). They can also reframe it to arrive at a more integrated understanding of the experience by identifying some redeeming features (Tedeschi and Calhoun, 2004). Indeed, following adversity, some individuals have reported positive change as a result of the experience (Park et al., 1996; Tedeschi and Calhoun, 1996). These include changes in sense of self (e.g., increased self-reliance and a sense that the experience “that did not kill you made you stronger”; Berger and Weiss, 2003, p. 28).

However, current literature on coping and meaning has often focused on major life events, while scant research has examined common daily adversities. Notwithstanding the significant role these major life events can play, they are relatively uncommon in a person’s lifespan (Serido et al., 2004; Frans et al., 2005). In contrast, common daily adversities, which can also disrupt core beliefs (O’Neill et al., 2004; Cann et al., 2011), and initiate meaning-making and growth are largely unexplored (Aldwin and Levenson, 2004; LoSavio et al., 2011). Though smaller in magnitude, negative daily events may disrupt people’s meaning systems particularly when they occur in valued life domains. For instance, negative daily social and achievement events are associated with less MIL on the day they occur (Machell et al., 2015). As such, we aimed to examine meaning-making processes in the context of negative daily experiences.

### 1.3. Positive reappraisal as a form of coping

Positive reappraisal is a form of meaning-focused coping which involves reinterpreting events or situations in a positive manner (Folkman and Moskowitz, 2000; Helgeson et al., 2006). It includes elements such as attempting to find benefits in the experience (Garnefski et al., 2001)—by searching for positive meaning among the negativity (Nowlan et al., 2015). This is in line with the concept of existential positive psychology, where meaning-focused coping strategies can promote a dialectical approach toward negative experiences (Wong et al., 2021)—by imbuing adversities with redeeming, positive features. The individual may come to believe something valuable or beneficial has been gained from the situation, such as enhanced wisdom or personal growth (Folkman and Moskowitz, 2000). Through reappraisal, people may come to believe that adversity has helped them to acquire wisdom and patience (e.g., Stainton and Besser, 1998); learn important life skills (e.g., Kimura and Yamazaki, 2013); appreciate the value of life (e.g., Chou et al., 2013); create a new sense of purpose by re-evaluating and identifying important values, relationships, and commitments (e.g., Park and Folkman, 1997); or test and thereby strengthen one’s faith and spirituality, and improve social relations (Cywińska, 2018).

As a coping strategy, positive reappraisal can be distinguished from primary stress appraisals such as the extent to which an event is important for one’s well-being and the potential for harm or growth (Lazarus and Folkman, 1984). Primary stress appraisals are particularly relevant in anticipation of demanding events (Peacock and Wong, 1990); in contrast, coping strategies are typically engaged in the aftermath of the event. How people cope with negative events depends in part on their perception of what can be done about it or its controllability (secondary appraisal; Lazarus and Folkman, 1984; Peacock and Wong, 1990). If the source of distress can be removed, one might engage in problem-focused coping and attempt to resolve the situation. If the situation cannot be resolved, one might engage in emotion-focused coping, which aims at reducing the negative affect associated with the stressful event. For example, a person might exercise or watch television to reduce their stress. Although positive reappraisal might be seen as a form of emotion-focused coping, Folkman (1997) (Folkman and Moskowitz, 2000) observed that by finding positive meaning in the experience, individuals more typically experienced higher levels of positive affect (PA) whether or not negative affect was mitigated. PA, in turn, has beneficial effects on coping resources and subsequent appraisals and coping efforts (Tugade and Fredrickson, 2004).

By facilitating PA, positive reappraisal may initiate a set of processes that further enhance meaning both by (i) broadening attention and improving meaning-readiness (e.g., King et al., 2006) and (ii) reducing the effects of NA and enabling more adaptive behaviors. Whereas dysfunctional and avoidant coping strategies can result in greater stress and worry (Ongaro et al., 2021), several studies show that inducing PA can facilitate greater engagement with negative experiences. For instance, recalling past acts of kindness led to less avoidance of negative information in an unrelated domain (Reed and Aspinwall, 1998). Success on an initial task also made participants more willing to examine weaknesses or failures on subsequent tasks (Trope and Pomerantz, 1998). More recently, a large-scale study (*N* = 12,243) found that meaning-centered coping was negatively associated with negative emotional states such as depression (including meaningless in life, low positive affectivity, and hopelessness), anxiety and stress during the COVID-19 pandemic (Eisenbeck et al., 2021). These findings suggest a feedback process in which PA and positive reappraisal reinforce each other while undoing the effect of NA on meaning-making.

Although positive reappraisal can be an effective coping strategy, recent studies suggest that the successful use of reappraisal requires several potentially taxing cognitive processes, including the ability to override a prepotent response (Ortner et al., 2016; Troy et al., 2018; Vieillard et al., 2020). For example, in emotionally intense situations, reappraisal may be difficult as it is challenging to override the original negative appraisal of the situation with the new, less emotionally evocative reappraisal (Ortner et al., 2016). Moreover, when given the choice to implement either reappraisal or distraction, participants were less likely to use reappraisal for high intensity emotional images (Sheppes et al., 2011, 2014; Shafir et al., 2015). The use of reappraisal is associated with decreased self-control resources when used in high-intensity situations (Sheppes and Meiran, 2008; Ortner et al., 2016); perhaps this explains why people are less likely to use reappraisal in such situations (Sheppes et al., 2014). An implication of these findings is that positive reappraisal may not be as functional in high-intensity situations where it could be most needed. Hence, it is important to find ways to facilitate the usage of positive reappraisal in these negative situations. One possibility is to attenuate the emotional intensity of the experience by altering one’s perspective on the event.

### 1.4. Effects of self-distancing on negative affect and meaning

Self-distancing is the process of stepping back from one’s own thoughts, beliefs, and feelings (Teasdale et al., 2002). Reflecting on adversity from a self-distanced perspective can facilitate constructive reasoning and effective regulation of negative emotions. It entails visualizing the event from a “fly on the wall” observer perspective—for example, by reflecting on it using third-person language (Grossmann and Kross, 2014; Kross and Ayduk, 2017; Nook et al., 2017). In contrast to the self-distanced perspective, people often adopt an egocentric view when focusing on past emotional experiences (Nigro and Neisser, 1983). This self-immersed (first-person) perspective draws attention to the concrete features of one’s experience (i.e., the specific course of events and emotions), thereby “reliving” the experience all over again (Robinson and Swanson, 1993; McIsaac and Eich, 2004). Indeed, when adopting a first-person perspective, individuals can experience high levels of emotional arousal, which may then hinder their ability to engage in cognitive analysis (Nigro and Neisser, 1983; Robinson and Swanson, 1993). However, a third-person perspective may draw attention to additional features of the situation—leading to appraisals that attenuate negative affect (Robinson and Swanson, 1993; McIsaac and Eich, 2004). This would allow individuals to focus on the broader context of the event and reconstrue their experience (Mischel et al., 1989; Trope and Liberman, 2003).

Individuals’ attempts to make meaning of a negative event often fail because they adopt a self-immersed perspective (Wang et al., 2019). However, when encouraged to think about the negative experiences from a psychologically distant perspective, individuals could reflect on them more constructively (Bruehlman-Senecal et al., 2016). This could be explained by the construal level theory which proposes that global and abstract construals are more inclusive than concrete construals, thus facilitating the inclusion of multiple stimuli into broader categories (Trope and Liberman, 2003, 2010; Schwarz and Bless, 2007; Förster et al., 2008). Because adopting a self-distanced perspective leads people to think about events in more abstract terms (Libby and Eibach, 2011)—and because abstraction accentuates the broader meaning of any given similarity—it is suggested that abstract levels of construals may facilitate meaning-making by making it easier to assimilate experiences into the global meaning system. Indeed, individuals who were able to envelop negative experiences (e.g., divorce, trauma)—into a broad understanding of their life’s narrative or into a broader explanatory framework—gained a sense that their lives have meaning (Pals, 2006; Bauer et al., 2008). Similarly, when people pictured an event from the third-person perspective, construed it abstractly, and integrated it with its broader context, they maintained a sense of meaning in the face of beliefs violations (Libby and Eibach, 2011). Thus, self-distancing could facilitate the assimilation of experiences into one’s global meaning system. By positioning the event within the grander scheme of one’s life, individuals may then arrive at a fuller understanding of the meaning of the event.

In addition to a main effect on situational meaning, self-distancing could potentially improve the effectiveness of positive reappraisal for enhancing meaning. This might be especially so when the negative experience is emotionally intense—requiring individuals to override an initially strong negative reaction with one that is more positive (Ortner et al., 2016). We hypothesized that positive reappraisal could be more effective in combination with a self-distanced perspective. By inviting a broader perspective on the event and reducing its emotional intensity, individuals may better identify positive implications from the experience—thereby enhancing the situational meaningfulness of the event.

### 1.5. The present study

As the meaning-making model suggests, global and situational meanings can both shape the present and future goals of a person as well as their overarching worldviews (Park, 2010)—analysis of one kind of meaning without the other would be incomplete. Indeed, scant research has investigated whether processes can jointly influence both global and situational meaning. In addition, despite the vital implications of daily experiences (O’Neill et al., 2004; Cann et al., 2011), little research has examined their effects on one’s sense of meaning (situational and global). Furthermore, given the multidimensional nature of meaning, the use of many generic and unidimensional measures may present oversimplified views of the underlying relationships between meaning and negative experiences (Martela and Steger, 2022). As such, a more nuanced approach is required to examine how each facet of meaning changes accordingly to meaning-making attempts.

To this end, the research goals are three-fold. First, we aimed to examine whether positive reappraisal and self-distancing—either in combination or alone—were effective approaches to enhancing meaning. Second, we explored whether the effectiveness of the meaning-making coping strategies on meaning would change according to the intensity of the negative experience. Third, we sought to investigate the extent to which meaning-making attempts influence individuals’ sense of situational meaning, global meaning, or both—across the three facets (i.e., coherence, purpose, and significance).

We designed a writing task following previous studies that have successfully manipulated either positive reappraisal and self-distancing. For example, participants who engaged in positive reappraisal reported more benefits from the experience such as finding a redeeming value in a loss of their loved ones (Folkman, 1997). In other studies, participants who were instructed to write using third-person (vs. first-person) pronouns reported more psychological distance and experienced fewer negative emotions (e.g., rejected, angry, and sad; Ayduk and Kross, 2010; Kross et al., 2011). Fewer studies have attempted to manipulate both positive reappraisal and self-distancing together. However, a recent study examining lower intensity cognitive interventions that target both affective (*via* self-distancing) and cognitive processes (*via* perspective broadening) found that, as compared to the control group, participants in the training condition reported lesser distress during the processing of negative experiences and reductions in residual symptoms of depression (Travers-Hill et al., 2017).

## 2. Methods

### 2.1. Participants

A total of 462 participants were recruited through the local university subject pool system (*M*_age_ = 21.28, *SD*_age_ = 1.91). The majority of the sample identified as female (78.7%; *N* = 364); 82.3% reported to be born in Singapore (*N* = 380); 79.9% identified Chinese as their ethnicity (*N* = 369). Table 1 summarizes the descriptive statistics of our sample. All participants received research participation credit upon completion of the study.

**Table 1:** Descriptive statistics of the key variables.

	Mean (SD)	Range	Kurtosis	Skewness
*Demographics*				
Age (in years)	21.29 (1.91)	18–32	3.45	1.27
Sex (% of females)	78%			
Born in Singapore (%)	82%			
Ethnicity (% of Chinese)	79%			
Religion (% of Christians)	32%			
Religion (% of Buddhists)	18%			
No religion (%)	33%			
*Individual differences*				
Dispositional gratitude	5.58 (0.88)	2.17–7	0.25	−0.68
Dispositional optimism	3.15 (0.69)	1–4.67	−0.49	−0.31
Global meaning	4.67 (0.93)	1.2–7	0.88	−0.40
Level of arousal	25.83 (6.27)	8–40	−0.54	−0.14
*Experience specific*				
Initial intensity	6.43 (1.26)	1–9	0.46	−0.14
Recency of the experience^1^	4.61 (2.00)	1–7	−0.92	−0.44
Resolution status^2^	4.06 (1.82)	1–7	−0.99	−0.20
Time to adopt the perspective (min)	0.31 (0.21)	0.04–1.04	0.76	1.10
Time to reflect (min)	4.47 (0.90)	3.02–5.70	−1.58	−0.21
Words written during reflection	128.71 (54.23)	16–325	0.48	0.76
Meaning in experience	4.19 (1.25)	1–7	−0.32	−0.15
Actual benefits accrued	41.02 (13)	9–63	−0.42	−0.47
Opportunity for benefits	36.22 (10.78)	8–56	−0.26	−0.39

1 Recency of the experience (i.e., memory age) is reported on a scale of 1 (Still ongoing) to 7 (Within the past 4 weeks).

2 Resolution status is report on a scale of 1 (not at all unresolved; not an active source of distress), 7 (very much unresolved; an active source of distress).

### 2.2. Procedure

The experiment conducted was a 2 (self-distanced vs. self-immersed) × 2 (positive reappraisal vs. reflection only) between-subjects design.

After providing informed consent, participants completed measures of dispositional optimism and dispositional gratitude—which served as covariates. They were then asked to identify a distressing/upsetting experience and write a sentence about it—before indicating their perceived initial intensity of experience. They were then assigned randomly to one of the four writing task conditions. After the writing task, participants completed a series of questions about their affective experience, psychological distance, perceived sense of benefits, situational meaning, and global meaning. Finally, they completed a set of demographic questions before debriefing (Table 2).

**Table 2:** Intercorrelations of the key variables.

Variables	1.	2.	3.	4.	5.	6.	7.	8.	9.	10.	11.	12.	13.	14.	15.	16.	17.	18.
1. Age (in years)	—																	
2. Dispositional gratitude	−0.04*	—																
3. Dispositional optimism	0.12*	0.38**	—															
4. Religiosity	−0.01	0.27**	0.29**	—														
5. Global meaning	0.16**	0.54**	0.51**	0.37**	—													
6. Level of arousal	0.12*	0.27**	0.27**	0.11*	0.3**	—												
7. Initial intensity	−0.01	0.00	−0.07*	−0.02	−0.08*	−0.01	—											
8. Recency of the experience	0.14*	0.09*	0.00	−0.08*	0.01	0.11*	0.09*	—										
9. Resolution status	0.01	−0.12*	−0.1*	−0.03	−0.08*	−0.06*	0.19**	−0.25**	—									
10. Time to adopt the perspective	0.02	−0.05*	−0.03	0.05*	−0.08*	0.03	−0.01	−0.01	−0.05*	—								
11. Time to reflect	−0.19**	−0.01	−0.05*	0.07*	0.01	0.04*	0.00	−0.09*	−0.06*	0.22**	—							
12. Words written during reflection	−0.14*	0.04*	−0.07*	−0.11*	0.00	0.03	0.09*	0.00	0.01	−0.02	0.38**	—						
13. Emotional reactivity	0.04*	0.01	0.02	0.02	0.1*	0.00	0.27**	−0.13*	0.39**	−0.01	0.1*	0.17**	—					
14. Post-reflection negative affect	0.06*	−0.13*	−0.1*	0.04*	−0.13*	−0.1*	0.00	0.00	0.33**	−0.06*	0.01	0.03	0.29**	—				
15. Post-reflection positive affect	0.1*	0.21**	0.17**	0.05*	0.28**	0.23**	−0.03	0.02	0.00	0.03	−0.03	0.01	0.11*	0.06*	—			
16. Meaning in experience	0.08*	0.19**	0.14*	0.14*	0.29**	0.1*	−0.02	0.00	0.04*	0.00	0.01	−0.04*	0.00	−0.02	0.4**	—		
17. Actual benefits accrued	0.14*	0.28**	0.23**	0.15**	0.34**	0.11*	−0.02	0.05*	0.00	0.01	0.08*	−0.04*	0.07*	0.00	0.45**	0.55**	—	
18. Opportunity for benefits	0.09*	0.25**	0.22**	0.24**	0.33**	0.15**	−0.02	0.04*	−0.01	0.03	0.13*	−0.04*	0.11*	0.02	0.45**	0.5**	0.83**	—

** *p* < 0.01;

* *p* < 0.05.

### 2.3. Materials and measures

#### 2.3.1. Writing tasks

Participants were instructed to think about a current or recent distressing or upsetting negative experience they are facing or have faced within the past 4 weeks. Then they wrote down a short anchor prompt to remind them of what the experience was about and rated the initial intensity of the experience (see below).^1^ Next, participants were randomly assigned to write about the experience from either a first-person (immersed) perspective or third-person (distanced) perspective. In addition, roughly half were instructed to simply recall the experience (reflection only) or to reconstrue it in a more positive manner (reappraisal). These two manipulations were crossed to create four experimental groups: immersed reflection (*n* = 113), distanced reflection (*n* = 110), immersed reappraisal (*n* = 119), and distanced reappraisal (*n* = 120). Each condition consisted of two parts that were 5 min each. Thus, all participants spent a total of 10 min on these tasks prior to evaluating situational and global meaning.

Participants in the reflection-only condition first worked on a *neutral task* where they answered a series of non-emotional questions related to the experience (e.g., “When did the experience occur? If possible, please include details such as date, day of the week, whether it was a weekday or weekend, the time of the day.”)^2^ for 5 min. They continued to work on the task until they finished all the questions or until the time was up. The purpose of this task was to extend the duration of the reflection-only condition so that it was roughly equal to the duration of the positive reappraisal condition. They then proceeded to a five-minute *reflection task* (Kross et al., 2012) where they recalled and analyzed their experience from either a self-distanced perspective (e.g., “Replay the experience as it unfolds in your imagination as you observe your distant self”) or self-immersed perspective (e.g., “Replay the experience as it unfolds in your imagination through your own eyes”). Participants in the distanced reflection (immersed reflection) condition were also instructed to write using third-person (first-person) pronouns to further draw the distinction between third-and first-person perspective (Giovanetti et al., 2019).

Participants in the positive reappraisal condition began by recalling their experience either through a self-distanced or self-immersed perspective for 5 min. Afterwards, they were prompted to think about the experience in a more positive light (Rood et al., 2012) for another 5-min. Specifically, they were instructed to give advice from either a self-distanced perspective (e.g., “Help the ‘distant you’ to see how they can benefit from … [the] experience …”) or a self-immersed perspective (e.g., “Help yourself to see how you can benefit from … [the] experience …”). Accordingly, they were instructed to write in either third-or first-person pronouns.^3^

#### 2.3.2. Initial intensity of experience

Following Rood et al. (2012) and Shiota and Levenson (2012), participants rated how they felt when they first went through the experience with the following three items: (1) severity of the event—“At that point of time, how bad did this experience feel like to you?”; (0 = *not bad/not terrible*, 8 = *the worst I have ever experienced*), (2) intensity—“At that point of time, how strong/intense were those emotions?”; (0 = *no emotion at all*, 8 = *the strongest emotions I have ever felt*), (3) valence—“At that point of time, how negative or positive did you feel?”; (0 = *very negative*, 8 = *very positive*). The first two items were averaged to form a single score for one’s initial intensity of the experience, where higher scores indicated a more intense event—the last item was dropped to improve reliability. The Spearman-Brown formula was used for reliability analysis for all two-items scales (Eisinga et al., 2013). Spearman-Brown coefficient for intensity of experience was 0.76.

#### 2.3.3. Task-induced mood

Using the 20-item Positive And Negative Affect Schedule (PANAS; Watson et al., 1988), participants indicated the extent to which they experienced Positive Affect (PA; e.g., “Interested,” “Enthusiastic”) and Negative Affect (NA; e.g., “Upset,” “Guilty”) while writing about the experience (1 = *very slightly or not at all*, 7 = *very much*). Items were summed to form a single score for PA (*α* = 0.92) and a single score for NA (*α* = 0.91).

#### 2.3.4. Psychological distance manipulation check

Adapted from Ayduk and Kross (2010) and White et al. (2015), participants indicated the extent to which they “were seeing [the event] through your own eyes versus watching it happen from a distance” (1 = *completely through my own eyes*, 7 = *completely from a distance*), and “how far away from [the event] did you feel” (1 = *very close*, 7 = *very far*). They were averaged to form a single score for psychological distance. The Spearman-Brown coefficient was 0.62.

According to McIsaac and Eich (2004), a self-immersed perspective is likely to direct individuals to narrowly focus on recounting the concrete, emotionally arousing details of their negative experiences—including the specific chain of events and the emotions felt. These details resemble the type of thought content that individuals who cognitively relive their negative experience tend to think about (Kross et al., 2005). In contrast, self-distancing allows individuals to analyze their thoughts and feelings from a broader perspective (Ochsner et al., 2004). As such, self-distancing should reduce the extent to which people focus on recounting the specific details of their negative experiences. In addition, reflecting in a self-distanced manner allows individuals to take the big picture into account and reconstrue their experience in a broader context (Kross et al., 2005). As such, adopting a self-distanced perspective is believed to encourage participants to engage in less recounting and more reconstruing. To evaluate these predictions, participants rated whether they *recounted* the specific chain of events that took place. They also rated three items asking whether they *reconstrued* the experience in ways that made them think and feel differently about their experience. Self-immersion is expected to increase recounting, whereas self-distancing increases reconstruing. Ratings were made on a 7-point scale (1 = *completely agree*, 7 = *completely disagree*). Three items on reconstruing were averaged to form a single score (*α* = 0.74). As the recounting scores and reconstruing scores were not negatively correlated, *p* = 0.98, they were not combined to form a single thought content score (Kross et al., 2005; Ayduk and Kross, 2008).

In addition, participants indicated the extent to which they were ‘reliving’ their recalled experience (Ayduk and Kross, 2010) with the following two items—“I re-experienced the emotions I originally felt during the experience when I think about it,” and “As I thought about the experience, my emotions and physical reactions to the experience were still pretty intense” (1 = *strongly disagree*, 7 = *strongly agree*). They were averaged to form a single score for emotion reactivity. Spearman-Brown coefficient for emotion reactivity was 0.78.

#### 2.3.5. Positive reappraisal manipulation check

Adapted from the Aspiration Index (AI; Kasser and Ryan, 1996) as well as the types of common benefits mentioned across the literature (e.g., Park and Folkman, 1997; Cywińska, 2018), participants rated (1 = *not at all*, 7 = *a great deal*) the extent to which reflecting and writing about the experience helped them realized nine accrued benefits (e.g., “Helped to clarify which goals or priorities are personally important and which are not”) and eight opportunities for benefits (e.g., “An opportunity for learning important life skills”). Items were added to form a single index for accrued benefits and a single index for opportunities for benefits.

#### 2.3.6. Situational meaning

Participants rated the meaningfulness of the experience using six items as adapted from Heintzelman and King (2014) and Waytz et al. (2015)—that assess situational meaning using the tripartite approach (1 = *not at all*, 7 = *very much*). Items include: *purpose* (“To what extent did the experience involve achieving a purposeful goal”; “To what extent was the experience full of purpose?”); *significance* (“To what extent did the experience make you feel significant”; “To what extent did the experience feel important rather than trivial?”); and *coherence* (“To what extent did the experience give you a sense of coherence”; “To what extent did the experience make sense?”). An additional item to indicate the overall judgment of meaningfulness of the experience was also included, “To what extent do you find the experience that you wrote about meaningless or meaningful” (−3 = *very meaningless*, 0 = *neither meaningful nor meaningless*, 3 = *very meaningful*). Spearman-Brown coefficients were calculated: purpose (0.76), significance (0.51), and coherence (0.65).

#### 2.3.7. Global meaning

Global meaning (i.e., MIL) was assessed with the 15-item Multidimensional Existential Meaning Scale (MEMS; George and Park, 2017). Participants rated (1 = *very strongly disagree*, 7 = *very strongly agree*) the extent to which they agreed with several statements assessing the facets of global meaning. They include, “I have aims in my life that are worth striving for” (purpose); “My life makes sense” (comprehension); and “I am certain that my life is of importance (mattering; 1 = *very strongly disagree*, 7 = *very strongly agree*). As adapted from Heintzelman and King (2014), an additional item to indicate the overall judgment of global meaning was also included, “To what extent do you feel that your life has meaning?” (1 = *not at all*, 7 = *extremely*). Three scores for purpose (*α* = 0.92), comprehension (*α* = 0.87), and mattering (*α* = 0.87) were calculated by averaging the five items within each subscale.

#### 2.3.8. Co-variates

As older memories are found to be more distant from the present than recent memories (Ayduk and Kross, 2010), participants also rated the recency of the experience (1 = *still ongoing*, 7 = *within the past four weeks*). Additionally, as the resolution of the recalled experience might have affected emotional reactivity (Ayduk and Kross, 2008), participants also rated the current status of the experience (1 = *not at all resolved*, 7 = *very much resolved*).

Individuals who hold generalized expectancies for positive outcomes (i.e., optimism; Scheier and Carver, 1985) may seek opportunities to transform threatening situations into favorable circumstances through positive reappraisal coping. Dispositional optimism was assessed with the six-item Life Orientation Test-Revised (Scheier et al., 1994; *α* = 0.81). Participants indicated the extent to which they agreed with the items (e.g., “Overall, I expect more good things to happen to me than bad”; 1 = *strongly disagree*, 5 = *strongly agree*).

Gratitude has been associated with making positive attributions, and a coping style called positive reinterpretation (Wood et al., 2008; Lambert et al., 2009). Dispositional gratitude was assessed with the six-item Gratitude Questionnaire-6 (McCullough et al., 2002; *α* = 0.80). Participants indicated the extent to which they agreed with the items (e.g., “I have so much in life to be thankful for”; 1 = *strongly disagree*, 7 = *strongly agree*).

## 3. Analytical strategy

One participant was manually screened out as due to noncompliance with the instructions (i.e., reflecting on an experience within the last 4 weeks). Prior to preliminary analyses, a robust outlier-detection approach was employed (i.e., minimum covariance determinant [MCD]; Leys et al., 2019)—which is based on median absolute deviation instead of the mean and standard deviation, as the latter can be considerably influenced by the outliers they were meant to identify. Outliers were detected based on three variables available in all conditions: (i) duration participants took to adopt either the self-immersed or self-distanced perspective, (ii) duration participants took to reflect on the experience, (iii) duration participants took to complete the study—these were chosen because a short duration may imply that participants did not reflect on the experience sufficiently, while a long duration may suggest that participants may not be focusing on the study. Using the MCD method with a breakdown point of 0.25 (i.e., computing the mean and covariance terms using 75% of the data; see Leys et al., 2019 for a discussion of this approach), 81 multivariate outliers were identified and removed; a final sample of 380 participants remained. Logistic regression was used to analyze the relationship between the conditions (self-distanced vs. self-immersed × reflection-only vs. reappraisal) on the probability of being an outlier: being randomly assigned to any of the four conditions did not significantly predict the probability of being an outlier, *p*s > 0.49. A series of independent samples *t*-tests further revealed that the outlier group tended to be less optimistic than the retained sample (*M*_retained_ = 3.15, *M*_outlier_ = 2.91, *t*(110.05) = 2.64, *p* = 0.01). In general, however, the outliers were not systematically different from the retained sample on the key variables. Subsequent analyses were conducted with and without these multivariate outliers. Assumptions of normality for all variables were then assessed. Values for skewness and kurtosis were within the acceptable standards for a normal distribution, that is, between −2 and +2 (George and Mallery, 2010). Table 2 displays the intercorrelations of the key variables involved in this study.

## 4. Results

### 4.1. Manipulation check

#### 4.1.1. Positive reappraisal

Two-way analyses of variance (ANOVAs) were performed to examine (i) perception of accrued benefits and (ii) opportunities for benefits between those in the positive reappraisal and reflection-only conditions. There was a main effect of positive reappraisal, *F*(1, 376) = 20.08, *p* < 0.01, no main effect of self-distancing, *F*(1, 376) = 0.16, *p* = 0.69, and no interaction between the positive reappraisal and self-distancing on accrued benefits, *F*(1, 376) = 0.07, *p* = 0.80. Similar results were obtained for opportunities for benefits: there was a main effect of positive reappraisal, *F*(1, 376) = 21.05, *p* < 0.01, no main effect of self-distancing, *F*(1, 376) = 1.21, *p* = 0.27, and no interaction between the positive reappraisal and self-distancing, *F*(1, 376) = 0.15, *p* = 0.70. Compared with those who only reflected on the event, those who reappraised reported more benefits (*M*s = 37.90 vs. 43.75), *t*(376) = 4.38, *p* < 0.01, *d* = 0.45, 95% CI [0.25, 0.66], and realized more opportunities for benefits (*M*s = 33.55 vs. 38.54), *t*(376) = 4.44, *p* < 0.01, *d* = 0.46, 95% CI [0.26, 0.67]. Thus, the positive reappraisal manipulation was successful.

#### 4.1.2. Self-distancing

Two-way ANOVAs were performed to examine the effectiveness of the self-distancing manipulation. Several measures were used to evaluate this.

##### 4.1.2.1. Psychological distance

There was a main effect of self-distancing, *F*(1, 376) = 24.19, *p* < 0.01, no main effect of positive reappraisal, *F*(1, 376) = 0.12, *p* = 0.73, and no interaction between positive reappraisal and self-distancing, *F*(1, 376) = 0.14, *p* = 0.71. Compared with immersed participants (*M* = 3.01), distanced participants felt more psychologically distant from the experience (*M* = 3.68), *t*(376) = 4.93, *p* < 0.01, *d* = 0.50, 95% CI [0.30, 0.71]. As expected, the self-distancing manipulation effectively created differences in psychological distance between the conditions.

##### 4.1.2.2. Thought content

There was a main effect of self-distancing on recounting, *F*(1, 376) = 5.42, *p* = 0.02, no main effect of positive reappraisal, *F*(1, 376) = 2.38, *p* = 0.12, and no interaction between positive reappraisal and self-distancing, *F*(1, 376) = 0.13, *p* = 0.72. Unexpectedly, immersed participants (*M* = 4.90) reported significantly less recounting than distanced participants (*M* = 5.25), *t*(376) = −2.33, *p* = 0.02, *d* = 0.24, 95% CI [−0.64, −0.05]. In addition, there was no main effect of self-distancing on reconstruing, *F*(1, 376) = 0.23, *p* = 0.63, and no interaction between positive reappraisal and self-distancing, *F*(1, 376) = 1.04, *p* = 0.31. A main effect of positive reappraisal was observed with those who reappraised the experience (*M* = 4.40) reconstruing it more than those who only reflected on the experience (*M* = 3.46), *F*(1, 376) = 51.64, *p* < 0.01, *d* = 0.74, 95% CI [0.53, 0.95]. Contrary to expectations, self-distancing did not lead to less recounting and more reconstruing.

##### 4.1.2.3. Emotional reactivity

There was no main effect of self-distancing, *F*(1, 376) = 0.46, *p* = 0.50, no main effect of positive reappraisal, *F*(1, 376) = 0.05, *p* = 0.83, and no interaction between positive reappraisal and self-distancing, *F*(1, 376) = 1.16, *p* = 0.28. Contrary to expectations, self-distancing did not lead to less emotional reactivity.

#### 4.1.3. Task-induced mood

For PA, there was a main effect of positive reappraisal, *F*(1, 376) = 29.09, *p* < 0.01, no main effect of self-distancing, *F*(1, 376) = 3.23, *p* = 0.07, and no interaction between positive reappraisal and self-distancing, *F*(1, 376) = 0.01, *p* = 0.92. Compared with participants who only reflected (*M* = 27.88), those who reappraised reported more PA (*M* = 34.69), *t*(376) = 5.36, *p* < 0.01, *d* = 0.55, 95% CI [0.34, 0.76].

For NA, there was a main effect of positive reappraisal, *F*(1, 376) = 21.19, *p* < 0.01, no main effect of self-distancing, *F*(1, 376) = 3.69, *p* = 0.06, and no interaction between positive reappraisal and self-distancing, *F*(1, 376) = 0.01, *p* = 0.92. Compared with participants who only reflected (*M* = 30.91), those who reappraised reported less NA (*M* = 24.92), *t*(351) = 4.58, *p* < 0.01, *d* = 0.47, 95% CI [0.27, 0.68].

The results of the manipulation checks suggested that the positive reappraisal manipulation was effective in eliciting perceptions of benefits, enhancing PA, and reducing NA. However, the effects of self-distancing manipulation were inconsistent. While it successfully created more psychological distance, the unexpected shift in thought content (e.g., more recounting) ran contrary to prior studies. The self-distancing manipulation did not reduce emotional reactivity and its effects on mood were not significant at the 0.05 level—although we note that mood tended to be less intense in the self-distancing condition (PA_Immersed_ = 32.56; PA_Distanced_ = 30.29; NA_Immersed_ = 28.99; NA_Distanced_ = 26.51). On the whole, these results suggest that the manipulation increased psychological distance from the experience but may not have activated other cognitive and affective processes reported in the self-distancing literature.

### 4.2. Hypothesis testing

#### 4.2.1. Effects on situational meaning

We conducted a moderated regression analysis with situational meaning scores regressed on positive reappraisal condition, self-distancing condition, initial intensity of the experience, and all two-way and three-way interaction terms among the three main predictors. Contrast coding was used to indicate which conditions participants were assigned to: the positive reappraisal (reflection only) condition were coded +1 (−1), and the self-distancing (self-immersion) condition were coded +1 (−1). Dispositional gratitude, dispositional optimism, recency of the experience, and resolution status were included in the regression model as control variables (Scheier and Carver, 1985; Lambert et al., 2009; Ayduk and Kross, 2010).

**Figure 1:**
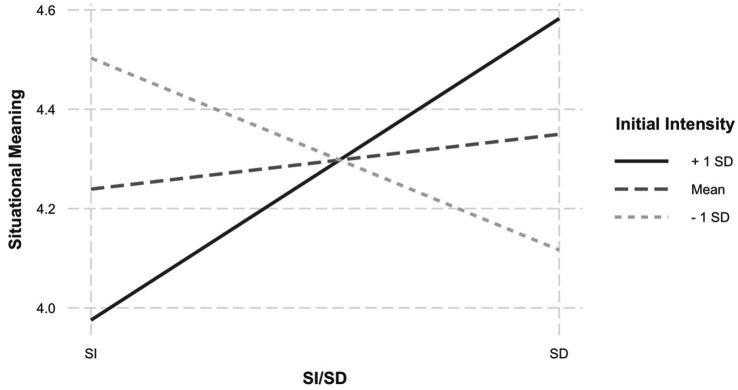
Initial intensity of negative experience moderates the effect of self-distancing on situational meaning. *Note*. SD, self-distanced reflection; SI, self-immersed reflection.

Results revealed a main effect of positive reappraisal on situational meaning, *b* = 0.44, *p* = 0.01, 95% CI [0.09, 0.78]—suggesting that engaging in positive reappraisal enhanced situational meaning (see Table 3). Although the main effect of self-distancing was not significant, a two-way interaction between self-distancing and initial intensity was observed, *b* = 0.46, *p* = 0.02, 95% CI [0.07, 0.84] (see Figure 1). To further probe the interaction, the Johnson-Neyman interval was obtained to determine the levels of intensity at which the simple slopes of self-distancing were significant. There was a significant negative relationship between self-distancing and situational meaning at lower levels of intensity (from 1.11 *SD* below the mean), and a significant positive relationship at higher levels of intensity (from 1.33 *SD* above the mean). In other words, self-distancing from low-intensity experiences reduced situational meaning compared with self-immersion. In contrast, self-distancing from high-intensity negative experiences enhanced situational meaning.

**Table 3:** Regression coefficients of the three-way interaction between positive reappraisal, self-distancing, and intensity on situational meaning.

Predictor	*b*	*t*	*p*
SD	0.08	0.48	0.63
PR	0.44	2.52	0.01
Intensity	−0.04	−0.19	0.84
SD × Intensity	0.46	2.34	0.02
PR × Intensity	−0.00	−0.02	0.98
SD × PR	−0.27	−1.58	0.11
SD × PR × Intensity	−0.33	−1.70	0.09

SD, self-distanced; SI, self-immersed; PR, positive reappraisal; RO, reflection-only.

#### 4.2.2. Facets of situational meaning

Given that meaning may be composed of distinct facets (George and Park, 2016, 2017; Martela and Steger, 2016), we further examined the effects of intensity, positive reappraisal, and self-distancing on each of these facets.

##### 4.2.2.1. Situational coherence

**Figure 2:**
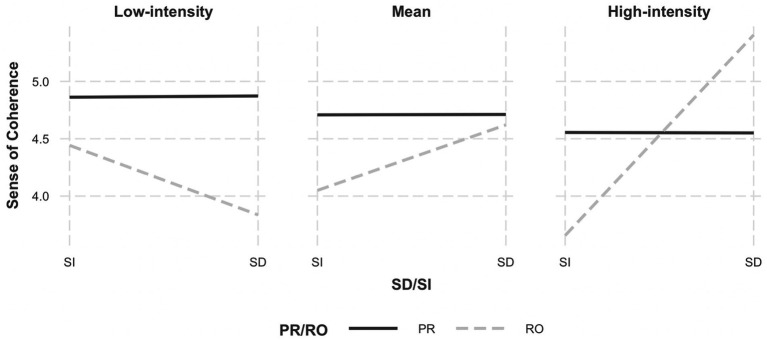
Initial intensity of negative experience moderates the interaction between positive reappraisal and self-distancing on situational coherence. *Note*. PR, positive reappraisal; RO, reflection-only; SD, self-distanced; SI, self-immersed.

Results revealed a main effect of positive reappraisal, *b* = 0.42, *p* < 0.01, 95% CI [0.19, 0.65] (Table 4). In addition, a non-significant main effect of self-distancing was qualified by a significant two-way interaction between self-distancing and intensity, *b* = 0.42, *p* = 0.04, 95% CI [0.02, 0.83]. Probing the interaction effect revealed only a significant positive relationship between self-distancing and coherence at higher levels of intensity (from 0.6 SD above the mean). These effects were further qualified by a three-way interaction between initial intensity, positive reappraisal, and self-distancing on situational coherence, *b* = −0.43, *p* = 0.038, 95% CI [−0.84, −0.02]. To interpret this three-way interaction, we first examined how the positive reappraisal × self-distancing interaction was moderated by the intensity of the negative experience (Figure 2). We observed that positive reappraisal × self-distancing interaction was significant at high-intensity experiences, *b* = −0.77, *p* = 0.03, 95% CI [−1.47, −0.06], but not at mean-level, *b* = 0.32, *p* = 0.25, 95% CI [−0.22, −0.85] or low-intensity experiences, *b* = −0.22, *p* = 0.22, 95% CI [−0.59, 0.14]. Among high-intensity experiences, the effects of positive reappraisal were similar whether it was performed with a third-person perspective (*distanced reappraisal*) or a first-person perspective (*immersed reappraisal*), *b* = 0.01, *t* = 0.02, *p* = 0.98 (the solid line in right panel of Figure 2). In contrast, when reflecting on the experience without positive reappraisal, taking a third-person perspective (*distanced reflection*) enhanced coherence more than taking a first-person perspective (*immersed reflection*), *b* = 1.54, *t* = 2.88, *p* < 0.01. In other words, the effects of self-distancing on coherence were only observed when reflecting without positive reappraisal—and only when reflecting upon high-intensity experiences. No effects of self-distanced reflection were observed at lower levels of intensity (mean-level: *b* = 0.47, *t* = 1.72, *p* = 0.09, low-intensity: *b* = −0.60, *t* = −1.45, *p* = 0.15).

**Table 4:** Three-way interaction between positive reappraisal, self-distancing, and intensity on situational coherence.

Predictor	*b*	*t*	*p*
SD	−0.00	−0.01	0.99
PR	0.42	3.55	<0.01
Intensity	−0.18	1.92	0.06
SD × Intensity	0.42	2.05	0.04
PR × Intensity	−0.15	−0.73	0.47
SD × PR	−0.22	−1.22	0.22
SD × PR × Intensity	−0.43	−2.09	0.04

SD, self-distanced; SI, self-immersed; PR, positive reappraisal; RO, reflection-only.

##### 4.2.2.2. Situational significance

Given the poor reliability of the two-item situational significance subscale, separate analyses were conducted for each item. The first item assessed the perceived importance of the experience (“To what extent did the experience feel important rather than trivial?”). The second item assessed existential mattering (“To what extent did the experience make you feel like your existence matters?”). When perceived importance was examined, only a main effect of positive reappraisal was observed, *b =* 0.27, *p* = 0.047, 95% CI [0.00, 0.54] (Table 5).

**Table 5:** Regression coefficients of the three-way interaction between positive reappraisal, self distancing, and intensity on perceived importance.

Predictor	*b*	*t*	*p*
SD	−0.05	−0.62	0.54
PR	0.27	2.37	0.047
Intensity	0.14	1.89	0.06
SD × Intensity	−0.02	−0.34	0.73
PR × Intensity	0.05	0.80	0.42
SD × PR	−0.02	−0.29	0.77
SD × PR × Intensity	−0.02	−0.29	0.77

SD, self-distanced; SI, self-immersed; PR, positive reappraisal; RO, reflection-only.

When existential mattering was examined, a main effect of positive reappraisal was also observed, *b* = 0.55, *p* < 0.01, 95% CI [0.21, 0.89] (Table 6). Qualifying this main effect was a significant a three-way interaction between initial intensity, positive reappraisal, and self-distancing, *b =* −0.62, *p* = 0.04, 95% CI [−1.20, −0.03]. We then examined how the positive reappraisal × self-distancing interaction was moderated by the intensity of the negative experience. The positive reappraisal × self-distancing interaction was significant at low-intensity, *b =* 0.60, *p* = 0.01, 95% CI [0.17, 1.37], and high-intensity experiences, *b =* −0.95, *p* = 0.049, 95% CI [−1.96, −0.07], but not significant at mean-level intensity, *b =* −0.17, *p* = 0.52, 95% CI [−0.69, 0.35].

**Table 6:** Regression coefficients of the three-way interaction between positive reappraisal, self-distancing, and intensity on existential mattering.

Predictor	*b*	*t*	*p*
SD	−0.21	−1.23	0.21
PR	0.55	3.16	<0.01
Intensity	−0.05	−0.34	0.73
SD × Intensity	−0.17	−1.21	0.23
PR × Intensity	0.21	1.52	0.13
SD × PR	0.10	0.60	0.55
SD × PR × Intensity	−0.62	−2.07	0.04

SD, self-distanced; SI, self-immersed; PR, positive reappraisal; RO, reflection-only.

**Figure 3:**
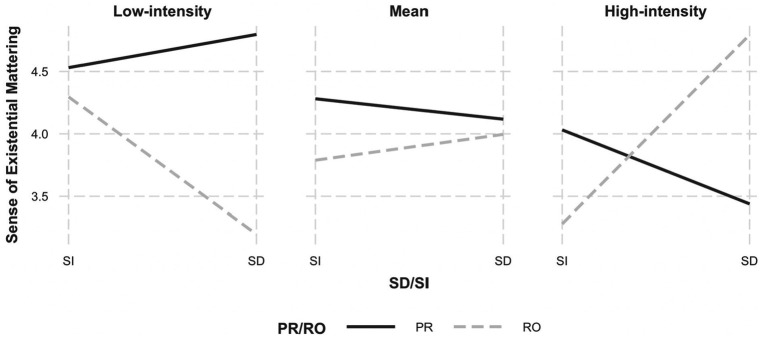
Initial intensity of negative experience moderates the effects of positive reappraisal and self-distancing on existential mattering. *Note*. PR, positive reappraisal; RO, reflection-only; SD, self-distanced; SI, self-immersed.

Among the low-intensity negative experiences, positive reappraisal had similar effects on mattering whether it was performed with a third-person perspective (*distanced reappraisal*) or a first-person perspective (*immersed reappraisal*), *b* = 0.25, *t* = 0.49, *p* = 0.62 (the solid line in left panel of Figure 3). In contrast, when reflecting on the experience without positive reappraisal, taking a third-person perspective (*distanced reflection*) reduced mattering more than taking a first-person perspective (*immersed reflection*), *b* = −1.45, *t* = −2.42, *p* = 0.02. Similarly, among high-intensity experiences, positive reappraisal had similar effects whether it was performed with a third-person perspective (*distanced reappraisal*) or a first-person perspective (*immersed reappraisal*), *b* = −0.32, *t* = −0.47, *p* = 0.64. However, when reflecting on high-intensity experiences without positive reappraisal, taking a third-person perspective (*distanced reflection*) enhanced mattering more than taking a first-person perspective (*immersed reflection*), *b* = 1.57, *t* = 2.03, *p* = 0.04. In other words, the effects of self-distancing on mattering—without positive reappraisal—depended on the intensity of the negative experience. While distanced reflection enhanced mattering at high intensities, it reduced mattering at low intensities.

##### 4.2.2.3. Situational purpose

The only effect to emerge from this analysis was the main effect of positive reappraisal, *b* = 0.60, *p* < 0.01, 95% CI [0.31, 0.90] (Table 7). No other effects were observed.

**Table 7:** Regression coefficients of the three-way interaction between positive reappraisal, self-distancing, and intensity on situational purpose.

Predictor	*b*	*t*	*p*
SD	−0.07	−0.47	0.64
PR	0.60	3.99	< 0.01
Intensity	0.03	0.28	0.78
SD × Intensity	−0.17	−1.43	0.15
PR × Intensity	0.19	1.56	0.12
SD × PR	−0.03	−0.20	0.84
SD × PR × Intensity	0.05	0.44	0.66

SD, self-distanced; SI, self-immersed; PR, positive reappraisal; RO, reflection-only.

#### 4.2.3. Standard implementation of positive reappraisal and self-distancing

In the present study, the positive reappraisal and self-distancing manipulations were crossed with each other leading to a hybrid condition in which participants reappraised the negative experience from a distanced, third-person perspective (i.e., distanced reappraisal). Although the previous analyses focused on the main effects and interaction between these two manipulations, we felt it was also insightful to compare the standard implementation of these two approaches. Specifically, as positive reappraisal is usually performed from a first-person perspective, the immersed reappraisal condition represents how it is typically practiced. In contrast, as self-distancing is usually performed *without* reappraisal, the distanced reflection condition represents the more conventional approach. Thus, we revisited our analyses and compared the relative effects of immersed reappraisal and distanced reflection across the different measures of situational meaning. We constructed a 95% confidence interval [CI] for the predicted values of situational meaning for distanced reflection and immersed reappraisal and examined whether their CIs overlapped. If they do not overlap, then it is evident that the two means are significantly different at the *p* < 0.05.^4^

Although immersed reappraisal often produced higher levels of meaning than distanced reflection, there were two exceptions to this pattern. First, for high-intensity negative experiences, distanced reflection (95% CI [5.39, 6.09]) enhanced coherence more than immersed reappraisal (95% CI [4.22, 4.91]). In contrast, for low-intensity experiences, immersed reappraisal (95% CI [4.53, 5.20]) enhanced coherence more than distanced reflection (95% CI [3.48, 4.22]).

A similar pattern was observed for existential mattering. For high-intensity negative experiences, distanced reflection (95% CI [4.80, 5.69]) enhanced mattering more than immersed reappraisal (95% CI [3.58, 4.45]). In contrast, for low-intensity experiences, immersed reappraisal (95% CI [4.10, 4.96]) enhanced mattering more than distanced reflection (95% CI [2.64, 3.57]). Broadly, these results suggest that for some aspects of meaning (i.e., coherence and mattering), self-distancing is more (less) effective than positive reappraisal for high (low) intensity experiences.

In sum, effects of positive reappraisal and self-distancing on situational meaning depended on the intensity of the experience. Specifically, engaging in self-distancing tended to enhance overall situational meaning, coherence, and mattering at high intensity but reduced them at low intensity. In contrast, positive reappraisal enhanced meaning across most indicators, but in two instances (coherence and mattering), this effect was qualified by intensity of the negative experience and whether reappraisal was performed in a self-distanced or self-immersed manner. A careful inspection of Figures 2, 3 indicates that the interaction effect has more to do with the effectiveness of distanced reflection than it does with positive reappraisal *per se*. Specifically, at low intensities, distanced reflection tended to *reduce* coherence and mattering relative to distanced reappraisal. However, at high intensities, distanced reflection *enhanced* coherence and mattering relative to distanced reappraisal.

#### 4.2.4. Effects on global meaning

In general, no effects of positive reappraisal or self-distancing were observed on measures of global meaning, nor were their effects moderated by intensity. The only exception was a two-way interaction between self-distancing and initial intensity on sense of global purpose, *b =* 0.10, *p* = 0.03, 95% CI [0.01, 0.19] (Table 8). The pattern was similar to that of situational meaning (see Figure 1), whereby self-distancing significantly reduced global purpose for low (0.55 SD below the mean) but not high intensity experiences.

**Table 8:** Regression coefficients of the three-way interaction between positive reappraisal, self-distancing, and initial intensity on global purpose.

Predictor	*B*	*t*	*p*
SD	−0.06	−1.11	0.27
PR	0.00	0.06	0.95
Intensity	−0.03	−0.60	0.55
SD × Intensity	0.10	2.21	0.03
PR × Intensity	0.02	0.34	0.74
SD × PR	0.05	0.90	0.37
SD × PR × Intensity	−0.07	−1.54	0.12

SD, self-distanced; SI, self-immersed; PR, positive reappraisal; RO, reflection-only.

## 5. Discussion

The present study had three main aims. First, we examined the effectiveness of positive reappraisal and self-distancing for making meaning from negative events. Second, we explored the extent to which the intensity of the experience altered the effectiveness of the meaning-making coping strategies. Third, instead of relying on a unidimensional conceptualization of meaning, we investigated meaning at both levels (i.e., global, and situational), and across three facets (i.e., coherence, purpose, and significance).

On average, positive reappraisal enhanced overall situational meaning of negative experiences as well as specific facets (purpose, coherence, and mattering). Specifically, the main effects of positive reappraisal were statistically significant across nearly all indicators of situational meaning. However, in the case of coherence and mattering, these main effects were qualified by a three-way interaction between positive reappraisal, self-distancing, and the emotional intensity of the negative event.

Indeed, we expected a significant three-way interaction given our prediction that self-distancing would enhance the effectiveness of positive reappraisal for high-intensity negative experiences. However, the results revealed that the effects of positive reappraisal on coherence and mattering (the solid lines in the Figures 2, 3) were similar whether it was performed with a self-distanced (third-person) perspective or a self-immersed (first-person) perspective. This was true across levels of emotional intensity. Thus, our prediction was not supported.

When we carefully inspect the patterns underlying the three-way interaction (Figures 2, 3), we see that it is mainly driven by the effects of reflection *without* reappraisal (the dashed lines in the figures) and how it varies across different levels of intensity and perspectives (distanced versus immersed). For high-intensity negative events, distanced reflection enhanced coherence and mattering relative to immersed reflection. For low-intensity negative events, the effect was reversed: distanced reflection resulted in *less* coherence and mattering than immersed reflection. The three-way interaction emerges because these highly contrasting effects determine whether it is better to reappraise the negative experiences or to simply reflect on the experience from a distanced perspective. When experiences are emotionally intense, distanced reflection results in greater coherence and mattering than distanced reappraisal. When experiences are not very intense, distanced reflection actually results in less coherence and mattering than distanced reappraisal.

A possible objection is that “distanced reappraisal” (i.e., positively reconstruing the event from a third-person perspective) does not represent how positive reappraisal is typically practiced. Therefore, we also compared the standard implementations of positive reappraisal and self-distancing. That is, we compared reappraisal from the first-person perspective (*immersed reappraisal*) with reflection from a third-person perspective *without* reappraisal (*distanced reflection*). These analyses support the basic conclusion that when a negative event is highly emotionally charged, self-distancing is more effective than positive reappraisal for enhancing coherence and mattering. However, when a negative event is mild, positive reappraisal is more effective than self-distancing.

The discrepant results we obtain for self-distanced reflection at high and low emotional intensity may be surprising given past work has found that reflecting on a negative experience from a distanced perspective generally promotes situational meaning (Ayduk and Kross, 2008; Kross and Ayduk, 2008, 2009, 2011; Kross et al., 2014). A key difference between previous studies and ours is that the former tended to elicit very distressing life experiences (e.g., the loss of a loved one or divorce). In contrast, in the present study, participants were prompted for everyday negative experiences—such as problems in school (e.g., lack of cooperation from a group mate) or problems with relatives and family (e.g., argument with siblings). Thus, the events studied by Kross and colleagues tended to be of higher intensity, whereas the events elicited in the present study may have varied more across intensity levels.

Why might self-distancing reduce rather than enhance the meaningfulness of low-intensity experiences? Perhaps lower intensity experiences afford less complexity, with fewer insights emerging when broadening one’s perspective of the event. Alternatively, individuals may not have much personal investment or engagement in the low-intensity experiences to begin with (White et al., 2019), and thus, individuals are constrained by the amount of content and information they can work with when they engage in self-distancing—inducing a floor effect.

Finally, whereas positive reappraisal and self-distancing affected situational meaning in various ways, no effects emerged for global measures of meaning (with the exception of global purpose). Perhaps this is not surprising given that only a single event—and one that could be relatively mundane—was examined. Nevertheless, it is possible that if individuals were trained to repeatedly process their negative experiences by engaging in reappraisal or self-distancing, a cumulative effect on global meaning could emerge over time. Future research to examine such interventions with experience sampling methodology would be extremely insightful.

### 5.1. Implications

In line with previous studies, our findings indicate that the tendency to engage in a self-immersed reflection (i.e., without positive reappraisal) results in diminished sense of meaning (Ayduk and Kross, 2010). In the attempt to understand the negative experience, individuals often engage in rumination. This perpetuates their fixation on self-relevant negative content—and may subsequently reduce their sense of meaning. However, we found that either adopting a self-distanced perspective or engaging in positive reappraisal buffered individuals against the reduced levels of meaning after a negative experience. Importantly, the effectiveness of one approach versus the other depends on (i) the emotional intensity of the experience; as well as (ii) which component of meaning one seeks to enhance.

Although positive reappraisal is generally an effective meaning-making strategy across a range of negative experiences, it may not be the most effective strategy for those that are highly emotionally charge. In particular, if individuals are struggling to make sense of such experiences or are questioning whether their own existence has value—reflecting on the experience in a distanced manner might be more helpful than attempting to reappraise it in a more positive manner. This observation could improve the development of meaning-based intervention. For example, expressive writing tasks could be structured in specific ways to promote one’s sense of meaning rather than simply divulging one’s deepest thoughts and feelings. Instead of delineating the concrete terms of the experience, a self-distanced reflection of the experience could foster additional insights and closure. Moreover, engaging in these writing tasks in the form of either positive reappraisal, or distanced reflection in one’s daily life is both time-and cost-efficient to make meaning out of negative events. Other approaches that seek to alter negative emotional responses through self-compassion may also help people develop new meanings from daily negative experiences (e.g., Sebri et al., 2022). Therefore, applying strategies that seek to reduce NA as well as promote an alternate perspective of the negative experiences may be especially critical in promoting meaning in long-term distressing circumstances such as COVID-19 pandemic (Eisenbeck et al., 2021; Wong et al., 2021).

### 5.2. Limitations and future research

The self-distancing manipulation may have exerted a small effect and thus be ineffective in certain ways. For example, distanced participants did not differ significantly from immersed participants in their emotional reactivity—as assessed by the extent to which participants re-experienced the negative emotions they felt in the original event. This is inconsistent with prior studies (e.g., Kross et al., 2005; Ayduk and Kross, 2008; Kross and Ayduk, 2009). One factor could be the type of emotion elicited by the negative experience. Prior studies instructed participants to write about specific experiences (e.g., one that elicited anger or sadness). In contrast, the present study simply instructed participants to write about a negative experience. Hence, other negative emotions such as guilt and shame could also be evoked—which self-distancing may not be as effective in regulating (Katzir and Eyal, 2013). Further, although distanced participants reported lower levels of NA and PA on average than immersed participants, this difference was not statistically significant. It is important to note that affect was measured using the PANAS, which mainly consists of adjectives representing high activation and arousal (Jovanović, 2015; Jovanović et al., 2019). Affect characterized by low to medium arousal may not be adequately measured using PANAS. Thus, future research should consider measures that fully capture the diversity of positive and negative feelings, across varying arousal levels (for a review see Tov et al., 2023).

We also recognize that the implications and generalizability of this study are limited by the use of a predominately female Singaporean student sample. While it is not the primary aim of the study to examine the moderating role of gender, recent studies have revealed gender differences vis-à-vis positive reappraisal. Specifically, positive reappraisal was negatively associated with depressive symptoms more so in females than in males (Duarte et al., 2015). In contrast, we found that positive reappraisal strengthened the perception of benefits in females and increased greater positive affect in males (Footnote 3). Thus, future research should consider the role of gender when developing meaning-based interventions.

In addition, cultural differences in the effects of positive reappraisal and self-distancing can be explored. A recent study suggested that dialecticism—the assumption that contradictory information can coexist (Peng and Nisbett, 1999)—may influence the ability to appraise negative situations more positively (Chen and Lee, 2021). For instance, East Asians (higher in dialecticism) were able to focus more on the positive aspects of negative events, as compared to North Americans who tended to hold more polarizing attitudes (Peng and Nisbett, 1999; Grossmann et al., 2014). Hence, individuals endorsing high dialectical thinking may face lesser resistance and difficulty in engaging positive reappraisal—as it involves the integration of positives (i.e., perceived valued gains) with the negatives (i.e., distressing reality). This could explain why we did not find an additional benefit of reappraising negative experiences from a distanced perspective, given our Singaporean sample. It is possible that in cultures where positive reappraisal may be more difficult, its effectiveness could be aided by practicing it from a third-person perspective.

## Data availability statement

The datasets presented in this study can be found in online repositories. The names of the repository/repositories and accession number(s) can be found below: https://osf.io/s93hz.

## Ethics statement

The studies involving human participants were reviewed and approved by Singapore Management University Institutional Review Board. The patients/participants provided their written informed consent to participate in this study.

## Author contributions

CL and WT contributed to the conceptualization, design, and implementation of the research, to the analysis of the results, and to the writing of the manuscript. WT supervised the project. All authors contributed to the article and approved the submitted version.

## Conflict of interest

The authors declare that the research was conducted in the absence of any commercial or financial relationships that could be construed as a potential conflict of interest.

## Publisher’s note

All claims expressed in this article are solely those of the authors and do not necessarily represent those of their affiliated organizations, or those of the publisher, the editors and the reviewers. Any product that may be evaluated in this article, or claim that may be made by its manufacturer, is not guaranteed or endorsed by the publisher.

## Footnotes

^1^The events that the participants wrote about fell into several broad categories: 143 (31%) involved important problems at school, 94 (20%) involved important problems with relatives and family, 85 (18%) involved fights among or with friends, 52 (11%) involved relationship problems, 40 (9%) involved important problems at work, and the remaining 48 (8%) were unclassified events (e.g., health conditions such as surgery, knee injury). Chi-square analysis suggested no group differences between the four experimental conditions, *χ*^2^(15) = 11.108, *p* = 0.74.

^2^These questions were designed on an *ad-hoc* basis for this study.

^3^We conducted additional analyses to examine the effects of gender. The analyses revealed moderating effects of gender on positive reappraisal and self-distancing on the manipulation checks. The positive reappraisal manipulation induced more cognitive effects (e.g., perceived benefits of the experience) for females than males. However, the manipulation induced more positive affect in males than in females. The self-distancing manipulation was generally effective but for males, greater psychological distance was only achieved when self-distancing was performed without positive reappraisal (see Supplementary materials for more details). No other moderating effects were observed. Further, including gender as a covariate in all analyses did not alter the results substantially.

^4^It has been suggested that looking at the overlap of the 95% CI of the means between two groups is not comparable to examining whether the two means are significantly different from each other at *p* < 0.05 (e.g., Austin and Hux, 2002; Knol et al., 2011)—as the former tended to produce a probability of a Type 1 error below 0.05 (i.e., 0.0056), which is a more conservative estimate. As such, it is recommended to use the criterion of an 83% CI overlap to determine whether two means are significant different from each other at *p* < 0.05. However, the results were similar when using either 95% or 83% CI.
