# Supplementary material for: Effects of positive reappraisal and self-distancing on the meaningfulness of everyday negative events
Source: Front Psychol. 2023 Feb 15;14:1093412. doi: 10.3389/fpsyg.2023.1093412 (PMC10034987; doi:10.3389/fpsyg.2023.1093412)
Supplement: Supplementary file 1 [file Data_Sheet_1.pdf]

frontiers

# *Supplementary Material*

## 1 Moderating effects of Gender

### 1.1 Accrued Benefits

A two-way interaction between positive reappraisal and gender on perception of accrued benefits was found, $F(1, 366) = 5.63$, $p = .02$ (see Figure 1). The effect of positive reappraisal on perception of accrued benefits was significant for females ($n = 300$), $t(366) = 4.82$, $p < .01$, but not males ($n = 74$), $t(366) = 0.51$, $p = .96$.

### 1.2 Opportunities for Benefits

A two-way interaction between positive reappraisal and gender was found on perception of opportunities for benefits, $F(1, 366) = 4.20$, $p = .041$ (see Figure 2). The effect of positive reappraisal on perception of opportunities for benefits was significant for females, $t(366) = 5.93$, $p < .01$, but not males, $t(366) = 0.09$, $p = .99$.

### 1.3 Psychological Distance

A three-way interaction between positive reappraisal, self-distancing, and gender on psychological distance was found, $F(1, 366) = 5.85$, $p = .02$ (see Figure 3). Simple interaction analyses revealed the two-way interaction effect between positive reappraisal and self-distancing was significant for males, $F(1,70) = 6.37$, $p = .01$, and not significant for females, $F(1, 296) = 0.20$, $p = .65$. Among the male participants, the effect of self-distancing on psychological distance was significant for those in reflection-only condition, $t(70) = 3.12$, $p = .01$, and not significant for those in the reappraisal condition, $t(70) = 0.34$, $p = .98$.

### 1.4 Recounting

A three-way interaction between positive reappraisal, self-distancing, and gender on recounting was found, $F(1, 366) = 4.78$, $p = .03$ (see Figure 4). Simple interaction analyses revealed the two-way interaction effect between positive reappraisal and self-distancing was significant for males, $F(1,70) = 4.45$, $p = .04$, and not significant for females, $F(1, 296) = 1.12$, $p = .29$. Among the male participants, the effect of self-distancing on recounting is significant for those in reappraisal condition, $t(70) = 2.14$, $p = .04$, and not significant for those in the reflection-only condition, $t(70) = -0.90$, $p = .37$.

### 1.5 Task-induced Positive Affect

A two-way interaction between self-distancing and gender was found on task-induced positive affect (PA), $F(1, 366) = 5.00$, $p = .026$ (see Figure 5). The effect of self-distancing on task-induced PA was significant for males, $t(366) = 3.12$, $p < .01$, but not females, $t(366) = 1.02$, $p = .31$.

## 2 Supplementary Figures

**Figure 1** *Gender Moderates the Effect of Positive Reappraisal on Perception of Accrued Benefits*

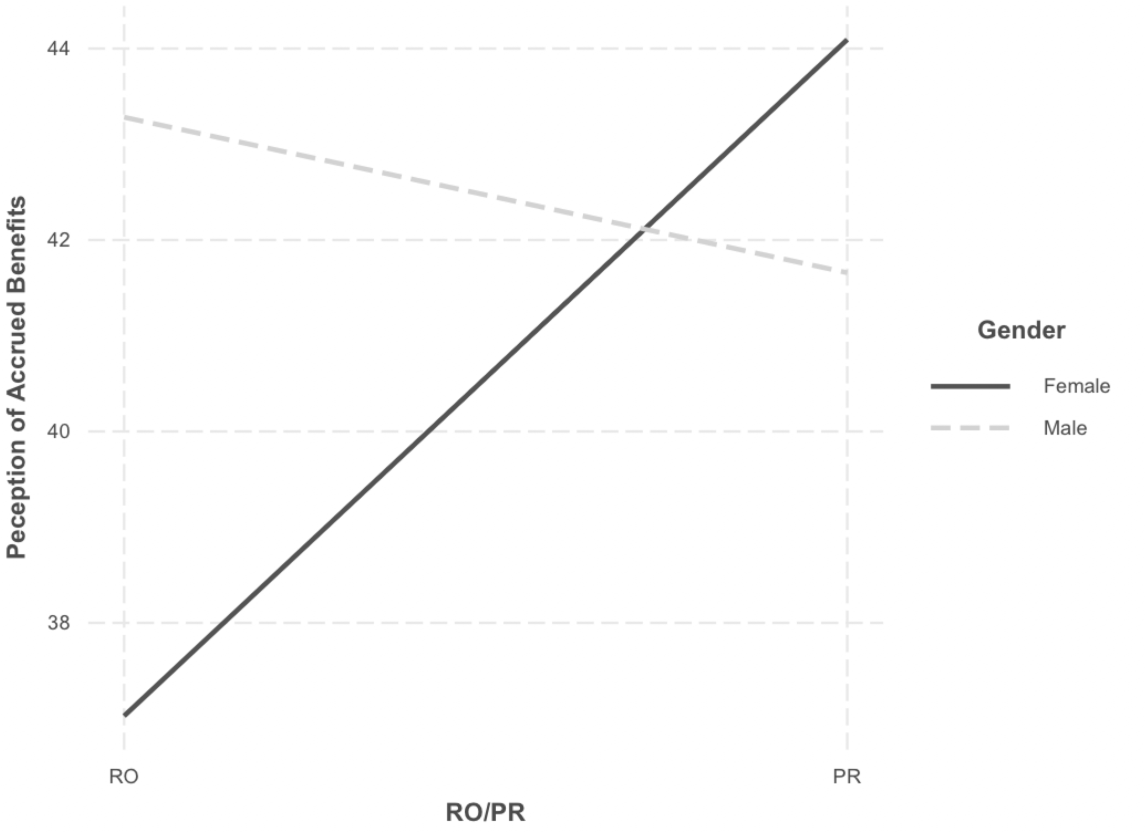


*Note*. PR = Positive Reappraisal; RO = Reflection-only.

**Figure 2** *Gender Moderates the Effect of Positive Reappraisal on Opportunities for Benefits*

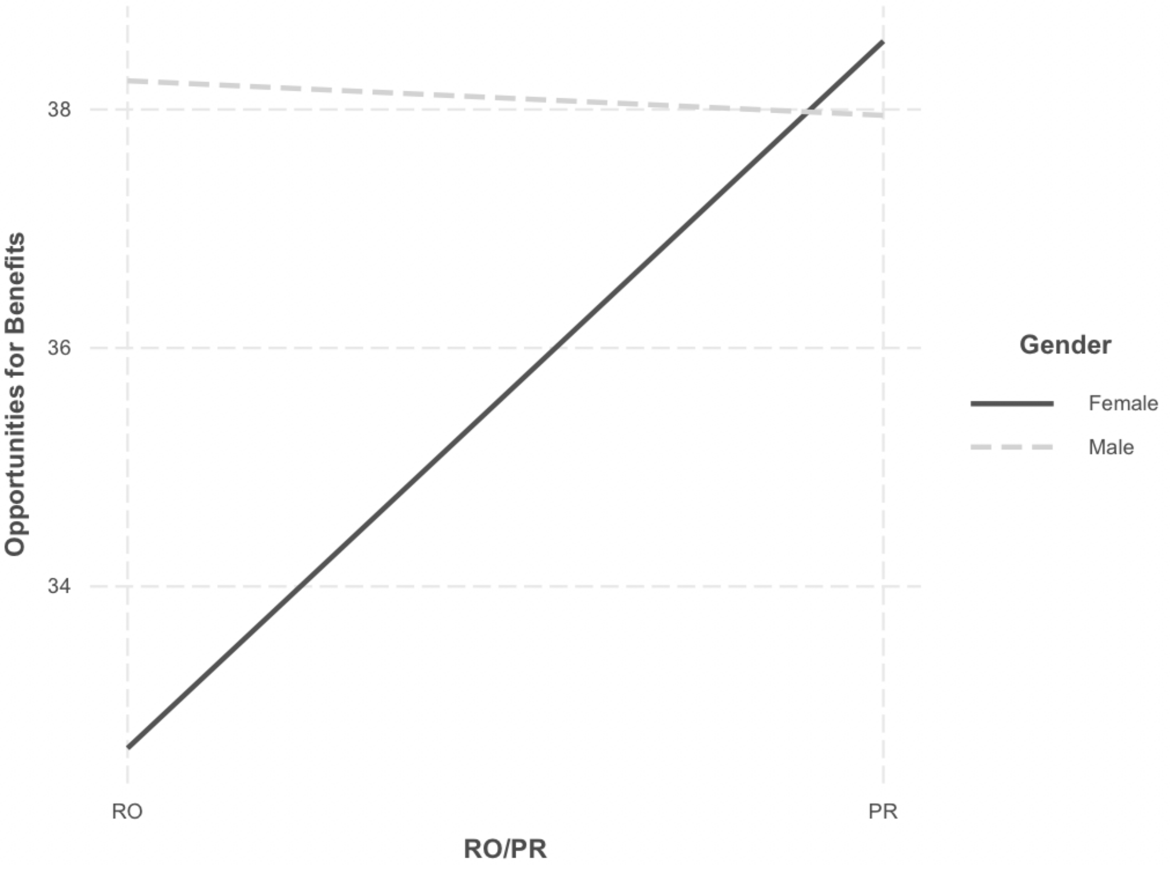


*Note*. PR = Positive Reappraisal; RO = Reflection-only.

**Figure 3** *Gender Moderates the Interaction between Positive Reappraisal and Self-Distancing on Psychological Distance*

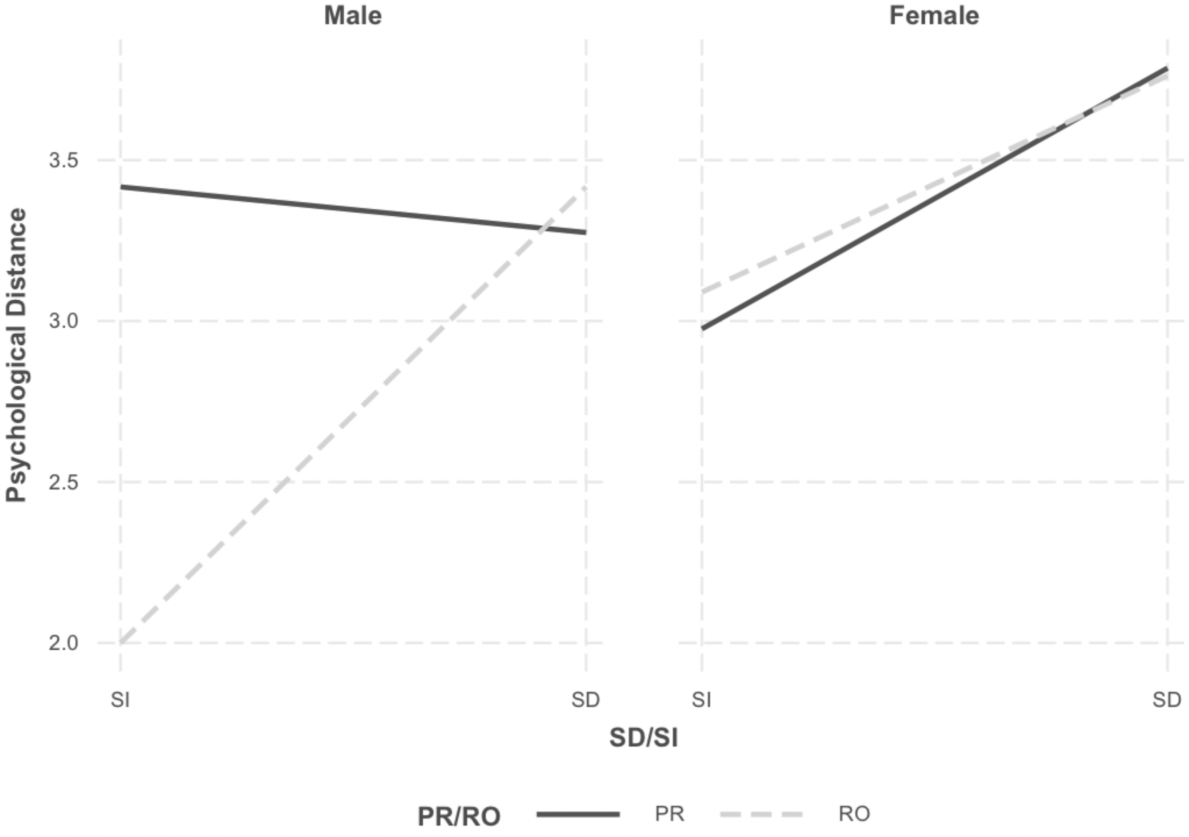


*Note*. SD = Self-distanced; SI = Self-immersed; PR = Positive Reappraisal; RO = Reflection-only.

**Figure 4** *Gender Moderates the Interaction between Positive Reappraisal and Self-Distancing on Recounting*

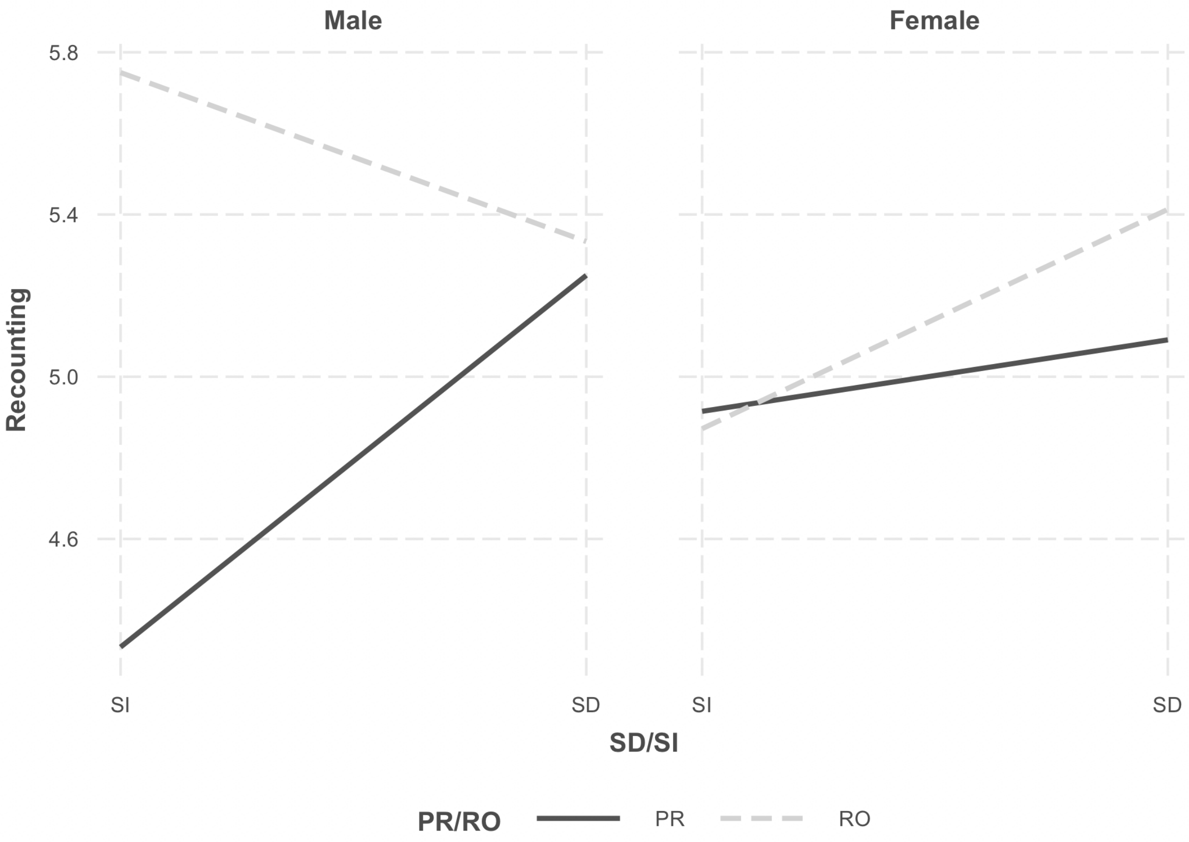


*Note*. SD = Self-distanced; SI = Self-immersed; PR = Positive Reappraisal; RO = Reflection-only.

**Figure 5** *Gender Moderates the Effect of Positive Reappraisal on Task-induced Positive Affect*

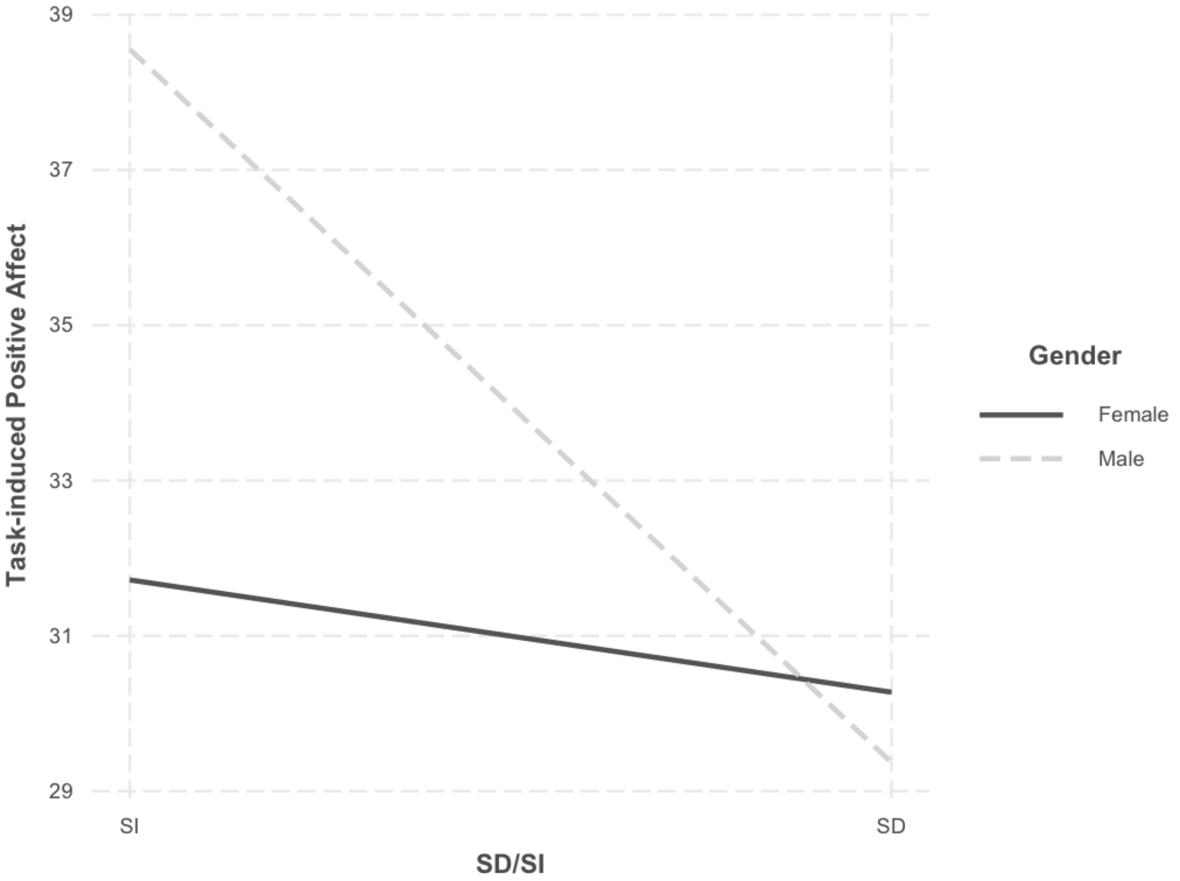


*Note.* SD = Self-distanced reflection; SI = Self-immersed reflection.
